# Monitoring the genetic diversity in holothurian populations from the Italian coasts with mitochondrial DNA sequences

**DOI:** 10.1038/s41598-024-76087-5

**Published:** 2024-10-19

**Authors:** Anisa Ribani, Valeria Taurisano, Arnold Rakaj, Alessandra Fianchini, Luca Grosso, Davide Pensa, Domitilla Pulcini, Luca Buttazzoni, Giuseppina Schiavo, Samuele Bovo, Francesca Bertolini, Valerio Joe Utzeri, Fausto Tinti, Fabrizio Capoccioni, Luca Fontanesi

**Affiliations:** 1 Animal and Food Genomics Group, Division of Animal Sciences, Department of Agricultural and Food Sciences, University of Bologna, Viale Giuseppe Fanin 46, 40127 Bologna, Italy; 2 Laboratory of Experimental Ecology and Aquaculture, Department of Biology, University of Rome Tor Vergata, Rome, Italy; 3 Centro di ricerca “Zootecnia e Acquacoltura”, Consiglio per la ricerca in agricoltura e l’analisi dell’economia agraria (CREA), 00015 Monterotondo (Rome), Italy; 4 Department of Biological, Geological, and Environmental Sciences, University of Bologna, Via S. Alberto 163, 48123 Ravenna, Italy

**Keywords:** COI mitochondrial gene, Genetic diversity, *Holothuria*, Italian coasts, Sea cucumbers, Genetics, Zoology

## Abstract

Sea cucumbers are deposit feeding members of marine benthic communities. The over-exploitation of sea cucumber natural stocks, especially in the Mediterranean basin, is having negative impacts on the marine ecosystem. This concerns had led the Italian government to take legal actions to preserve these important marine resources. The aim of this study was to evaluate the level of genetic diversity and population structures within two sympatric *Holothuria* sea cucumber species, *H. polii* and *H. tubulosa*, across ten Mediterranean areas along Italy. A 323 bp portion of the COI mitochondrial gene was sequenced in a total of 441 holothurians (251 *H. polii*, 177 *H. tubulosa* and 13 from other species). Genetic diversity analyses and analysis of molecular variance (AMOVA) suggest that *H. polii* and *H. tubulosa* species are distinct, but within and among populations are homogeneous, indicating active gene flows across the Mediterranean areas investigated. *H. polii* showed a lower genetic diversity than *H. tubulosa*, probably related to differences in life history traits. Phylogenetic analyses showed a clear differentiation between the two species, even if six specimens morphologically assigned to a species clustered within the other species, indicating a possible occurrence of hybridization events. These data will be useful in implementing conservation actions for these holothurian genetic resources.

**Supplementary Information:**

The online version contains supplementary material available at 10.1038/s41598-024-76087-5.

## Introduction

Holothurians (Holothuroidea, de Blainville 1834), commonly known as sea cucumbers, are a class of marine invertebrates belonging to the Echinodermata phylum. Sea cucumbers are benthic deposit feeders that ingest marine sediment selecting organic matter with their tentacles, including vegetal and animal detritus, bacteria, protozoa and diatoms^1,2^. As detritivores, sea cucumbers play a key role in seabed dynamics by processing and bioturbating sediments^3^.

Holothurians have unique defence strategies: they contain in their body wall substances toxic to some other species, they can expel Cuvierian tubes or eviscerate their digestive tract and respiratory trees. The expelled organs can then be regenerated in a very short time^4^. The body wall of these organisms contains many bioactive compounds that are considered to have high potential in Asian traditional medicine^5^. Many holothurian species are economically important as luxury food primarily in Asian markets. Therefore, the increased consumption of these species has led to the over-exploitation of many natural stocks, first in Asia and then in other regions of the world^6^. Overfishing has led to the collapse of natural stocks and, in some regions, the complete extinction of holothurians, with a negative impact on marine ecosystems^3,7,8^. For example, Clements et al.^9^ have evidenced that in coral reef ecosystems where sea cucumber communities are removed coral diseases and mortalities are ~ 1500% higher than in unexploited areas^9^. For these reasons, multiple initiatives are underway to explore the potential of sea cucumbers as promising aquaculture species. In this context, cultivating these invertebrates through aquaculture could offer an alternative solution to meet current market demands and help with restoring local wild populations^2,10,11^. Additionally, certain holothurians have been effectively utilized in integrated multitrophic aquaculture (IMTA) systems, leveraging their trophic behaviour to recycle organic waste from fish farms^12,13^.

*Holothuria polii* (Delle Chiaje, 1823) and *H. tubulosa* (Gmelin, 1788) are Mediterranean endemic species. They typically inhabit the sublittoral zone at depths ranging from 1 to 150 m for *H. tubulosa*^14^ and up to 250 m for *H. polii*^15^, often coexisting in seagrass meadows and on rich organic bottoms. Both *H. tubulosa* and *H. polii* reproduce in the summer, releasing large quantities of gametes into sea water where fertilization occurs^14^. Nevertheless, these two species exhibit different reproductive strategies, with *H. polii* showing lower fecundity (3.48 ± 1.41 vs. 0.176 ± 0.016 million of eggs) but larger offspring sizes (203.7 ± 10.21 μm vs. 151.2 ± 2.1 μm) than *H. tubulosa*^14^. The allocation of reproductive energy into a large versus small size or high versus a low number of offspring is an important life-history trait that may strongly affect larval development of these species and in turn their recruitment and population connectivity. In fact, *H. polii*, conversely to *H. tubulosa*, can be defined a facultative planktotrophic species, being able to metamorphose in benthic juveniles without any planktonic feeding^14^.

Despite differences in their life history traits, these sympatric species could have the potential to undergo hybridization events, even if there is no evidence of intermediate phenotypes between these two species^14^. Therefore, conducting DNA-based characterization can provide valuable information on the holothurian reproductive dynamics and genetic diversity within and among species^16^. Few population genetics studies have been conducted on *Holothuria* species: most of them analysed mitochondrial markers (primarily 16S and COI genes) or nuclear markers (both microsatellites and nuclear gene markers) resulting in high levels of haplotype diversity and low levels of nucleotide diversity among populations of different *Holothuria* species, including *H. polii* and *H. mammata*^4,7,17^.

Due to its well-known characteristics, mitochondrial DNA (mtDNA) genes as the COI mtDNA gene (the standard barcoding DNA region in animals) have been extensively used to investigate the population genetics and systematics in holothurian species^4,7,18–20^. This genetic marker has been recently investigated for the authentication of different sea cucumber products using a mini-barcoding approach with next generation sequencing technology, including *H. polii* and *H. tubulosa* species^21^. The COI mtDNA gene has been considered an appropriate molecular marker for determining genetic diversity and assessing population genetic structure in *H. polii* and *H. tubulosa* populations, as reported by a few studies conducted in some Mediterranean areas^16^, in coastal lagoons of Mar Menor (Spain^20^) and along the Mediterranean Turkish coast^22^.

In the Mediterranean basin, especially along the Italian coasts, illegal and unregulated fishing of *H. polii* and *H. tubulosa* has dramatically increased in recent years. This led the Italian Ministry of Agriculture, Food Sovereignty and Forests (MASAF) to impose since 2018 an annual moratorium on fishing holothurians (Minister Decree n. 706727/2023^23^), applying the precautionary principle pursuant to art. 174 of the Amsterdam Treaty, establishing the prohibition of “fishing, holding on board, transhipping, or disembarking” of sea cucumbers, pending a scientific investigation aimed to provide information useful to support the adoption of structural measures for a sustainable management of these invertebrate species.

This study has been designed to provide an initial overview of the genetic structures and diversity of the *H. polii* and *H. tubulosa* populations along various Italian coasts. The specific aims were (i) to evaluate the genetic structures, assessing the holothurian population differentiations according to their geographical distribution; (ii) to evaluate if the COI mtDNA gene can serve as a marker for holothurian phylogeny and species identification and (iii) to determine any population dynamics and active gene flow among holothurian populations, testing positive or negative selection sweeps. Finally, the last goal is to offer decision makers insights to help define appropriate holothurian management strategies and prevent the erosion of these genetic resources.

## Methods

### Sampling

**Fig. 1:**
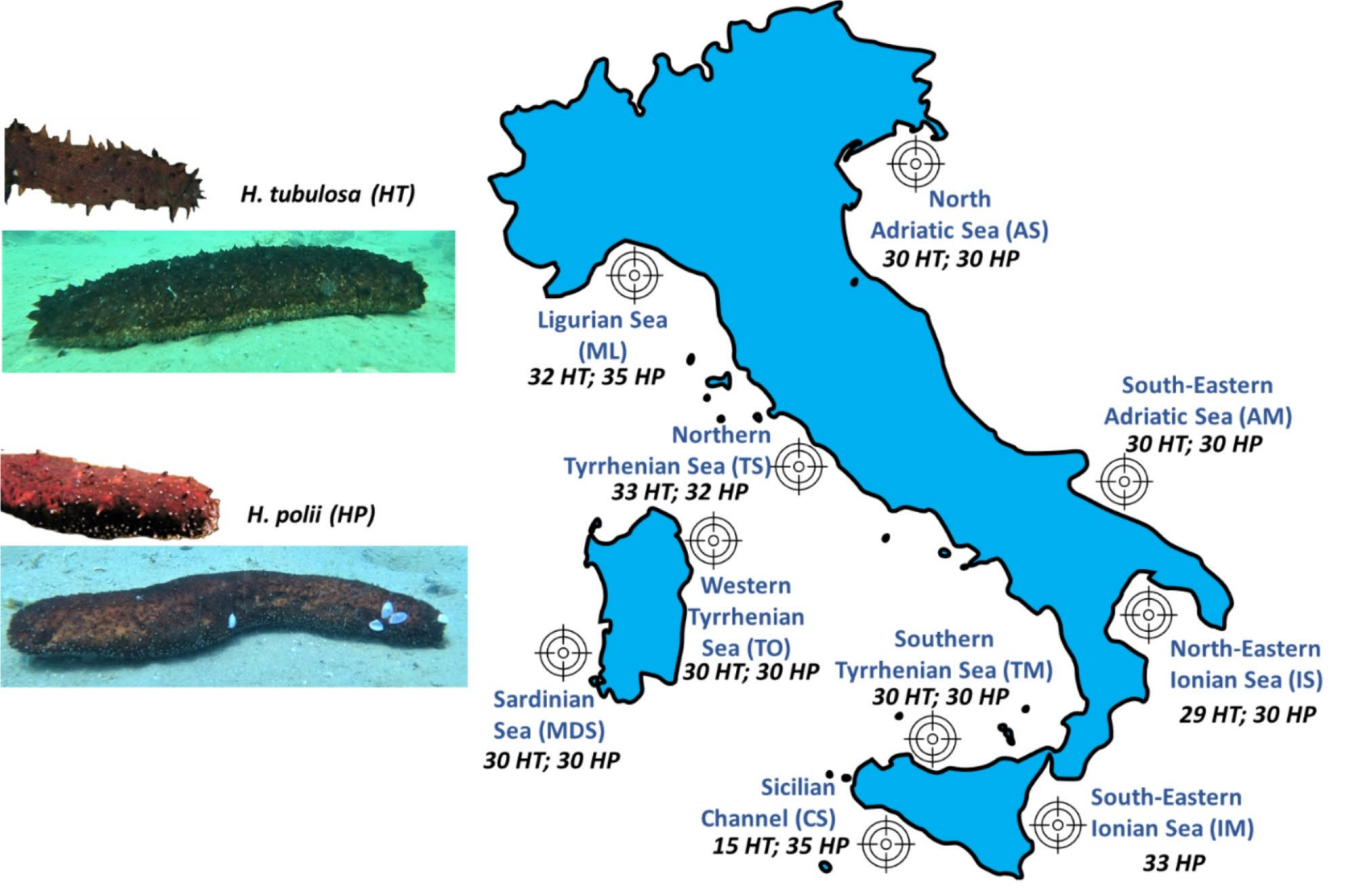
Sampling areas along the Italian coasts and number of specimens collected for *H. polii* (HP) and *H. tubulosa* (HT) populations. Map was generated with QGIS 3.30 (http://www.qgis.org, accessed on the 10th January 2024) and edited with GIMP v.2.10 tool (https://www.gimp.org/).

A total of 578 specimens, including 315 of *H. polii* and 259 of *H. tubulosa*, were collected from ten sampling areas located along the Italian coasts (from North-Est to South-West; Fig. 1). Each area was named in this study considering Italy in the middle of the geographical context: North Adriatic Sea (AS), South-Eastern Ionian Sea (AM), North-Eastern Ionian Sea (IS), South-Eastern Adriatic Sea (IM), Sicilian Channel (CS), Southern Tyrrhenian Sea (TM), Western Tyrrhenian Sea (TO), Sardinian Sea (MDS), Northern Tyrrhenian Sea (TS), and Ligurian Sea (ML). Specimens were collected at depths ranging from 8 to 22 m on sandy bottoms, seagrass meadows and mixed substrates. Additionally, two sea cucumbers of *H. polii* species, one specimen of *H. tubulosa* and one specimen of *H. mammata* were collected in the North Adriatic Sea nearby Rovinji (Croatia) at a depth of about 35 m. The number of *H. polii* (HP) and *H. tubulosa* (HT) specimens collected from each sampling sites is reported in Fig. 1.

### Morphological characterisation of the specimens

Species-specific morphological characteristics reported in the identification guidelines and dichotomic keys were used to assign collected individuals to the two species^14^. *H. tubulosa* is larger than *H. polii* and has a clear chromatic demarcation of the body wall, which is lighter on the ventral than on the dorsal side. On the other hand, *H. polii* is dark brown/black coloured with white tips on the ambulacral podia and papillae. Hence these morphological characters render these species clearly distinguishable from each other, conversely *H. tubulosa* is similar in size, shape, and colour to *H. mammata*, and distinction between them may be difficult. However, *H. tubulosa* can usually be distinguished from *H. mammata* by the clear demarcation in colour between the dorsal and ventral sides of the body wall, by its smaller and more slippery dorsal papillae, and by the absence of the Cuvierian tubules (which are present, albeit as a residue, in *H. mammata*).

### DNA extraction, PCR and sequencing

For each specimen, 40 mg of muscular bands was collected from single individual by dissection and preserved in a 1.5 mL tube with absolute ethanol. Samples were removed from ethanol and homogenized. Then, 1 mL of CTAB extraction buffer [2% (w/v) cetyltrimethylammoniumbromide; 2% (w/v) polyvinylpyrrolidone; 1.4 M NaCl; 100 mM Tris-HCl; 20 mM EDTA pH 8] was added to the homogenised sample along with 5 µL of RNase A solution (10 mg/mL) and 30 µL of proteinase K (20 mg/mL). After an incubation for 90 min at 65 °C with gentle mixing in a thermoblock instrument, samples were cooled to room temperature and then centrifuged for 10 min at 16,000 g. The supernatant was transferred in a new tube containing 500 µL of chloroform/isoamyl alcohol (24:1), vortexed for 20 s and then centrifuged for 15 min at 16,000 g. The upper phase of the supernatant was transferred to a new tube containing 500 µL of isopropanol for DNA precipitation; then, after an incubation at room temperature for 10 min, samples were centrifuged for 15 min at 16,000 g and the supernatant was discarded. The pellets were finally washed and purified by adding 500 µL of ethanol 70% and rehydrated with 50 µL of sterile water and stored at − 20 °C until PCR analyses. The concentration and quality of extracted DNA were measured using a nanophotometer IMPLEN P330 (Implen GmbH, München, Germany).

For PCR reactions, an ad hoc primer pair was designed using the Primer-BLAST tool of the National Center for Biotechnology Information (NCBI), having as target the holothurian COI mtDNA gene. The template for the PCR primer design was the *H. polii* mitochondrial reference genome (accession number LR694133^15^). The designed primer pair (forward: 5′-CCTCAGCAGGAGTAGAAAGAG-3′; reverse: 5′-CTCCAGCRGGGTCRAAGAAG-3′) amplifies a COI mtDNA gene fragment of 333 bp. The primers contain degenerated nucleotides in the polymorphic sites to amplify with the same efficiency orthologous gene fragment in *H. tubulosa*.

Amplifications were performed on a SimpliAmp Thermal Cycler (Thermo Fisher Scientific, Waltham, MA, USA). Polymerase chain reactions were carried out in a total volume of 20 µL including: KAPA HiFi HotStart Mastermix (Kapa Biosystems, Roche, Basel, Switzerland); 10 pmol of each primer; 10–50 ng of isolated DNA. The PCR profile followed these steps: initial denaturation step at 95 °C for 3 min; 35 cycles of alternate temperatures (20 s at 98 °C, 15 s at 59 °C, 30 s at 72 °C); a final extension step at 72 °C for 1 min. Amplified DNA fragments were electrophoresed on 2.5% agarose gels running in TBE 1X buffer and stained with 1X GelRed Nucleic Acid Gel Stain (Biotium Inc., Hayward, CA, USA).

PCR products were purified with ExoSAP-IT (USB Corporation, Cleveland, Ohio, USA) and then Sanger sequenced with the BrightDye^®^ Terminator Cycle Sequencing Kit (NIMAGEN, Nijmegen, The Netherlands). Sequencing reactions were loaded on an ABI3100 Genetic Avant capillary sequencer (Applied Biosystems, Foster City, CA, USA). All electropherograms were visually inspected and analysed with MEGA 11 software^24^.

### Phylogenetic and population genetic structure analyses

Newly generated COI mtDNA sequences were aligned with the COI mtDNA sequences of *Holothuria forskali* (accession no. NC_013884), *H. mammata* (ON059147), *H. scabra* (NC_027086), *H. arguinensis* (ON059144), *H. polii* (LR694133 and EU750824) and *H. tubulosa* (OP895104 and KY774361) retrieved from NCBI GenBank. The alignments of the sequences were conducted using the ClustalW algorithm implemented in MEGA 11 software. The Neighbour-Joining (NJ) phylogenetic tree was built based on Maximum Composite Likelihood (MCL) evolutionary distances with a Gamma distribution model of nucleotide mutation rate using 1000 bootstrap replicates^24–27^. Nodes in the tree with percentage of bootstrap replicates < 10% were collapsed. The phylogenetic tree was graphically edited using iTOL online software version 6.9 (https://itol.embl.de/^28^), based on the Tree of Life^29^. The *Ocnus glacialis* mitochondrial DNA sequence, retrieved from NCBI GenBank (NC_082941), was considered as outgroup for phylogenetic analysis.

Genetic diversity and population structure parameters were estimated between species, among and between populations using MEGA11^24^, DnaSP v6 (version 6.12.03)^30^ and Arlequin version 3.5.2.2 software^31^. The genetic diversity parameters have also been estimated in the two basins of the Mediterranean Sea to compare Italian Western and Eastern Mediterranean populations. The Italian Eastern Mediterranean area included populations collected in the North Adriatic Sea (AS), South-Eastern Adriatic Sea (AM), North-Eastern Ionian Sea (IS) and South-Eastern Ionian Sea (IM), while the Western area included populations from the Ligurian Sea (ML), Sardinian Sea (MDS), Northern, Western and Southern Tyrrhenian Sea (TS, TO, TM). Specifically, haplotype diversity, p-distance, nucleotide diversity (π) and pairwise F_ST_ index were calculated between *H. polii* and *H. tubulosa* species, among different populations and within populations. Gst and Gammast parameters were used to estimate gene flow and genetic differentiation^30^. Briefly, p-distance represents the proportion (p) of nucleotide sites at which two sequences being compared differ, calculated by dividing the number of nucleotide differences by the total number of nucleotides compared; haplotype diversity (*Hd*) represents the probability that two randomly sampled haplotypes are different; nucleotide diversity (π) is defined as the average number of pairwise nucleotide differences; the fixation index (F_ST_) is a measure of population differentiation due to genetic structure; Gst is the proportion of genetic diversity among populations and Gammast estimates the gene flow and the genetic differentiation^24,30,32^.

Furthermore, population expansion and bottlenecks were evaluated based on the Tajima’s^33^ and Fu’s Fs statistics^34^ using Arlequin version 3.5.2.2^31^. The Tajima’s D test compares the number of segregating sites per site with the nucleotide diversity π by calculating the Tajima’s D value. Tajima’s D compares the observed π against the expected diversity under the assumption that all polymorphisms are selectively neutral: negative Tajima’s D values signify an excess of low frequency polymorphisms relative to expectation and vice versa^31,33^. The Fu’s test was designed to detect an excess of rare alleles signifying recent population expansion^34^; given the size of the population (n), the observed number of alleles (k) and the nucleotide diversity (π), Fu’s Fs statistics calculates the probability of a random dataset with the same n and π, having at least as many as the observed k alleles: negative values of Fs are evidence for an excess number of alleles expected from a recent population expansion, while positive values of Fs is evidence for an deficiency of alleles expected from a recent population bottleneck^34^.

Both neutrality tests were performed on the entire dataset and within all populations, considering the echinoderm mitochondrial mutation rate in a coding sequencing and removing all ambiguous positions for each sequence pair, using the pairwise deletion option, using DnaSP v6 and Arlequin software^30,31,35^. Moreover, the neutrality tests were performed considering all nucleotide sites of the analysed COI mtDNA gene portion including all samples together or separately by species. The statistical significance for both tests was obtained assuming a Chi-square distribution with one degree of freedom, using DnaSP v6 and Arlequin software^30,31^.

Finally, analysis of molecular variance (AMOVA) was conducted considering four genetic structures to partition genetic variance among and within groups by computing conventional F-statistics from haplotype frequencies: (i) the two species separately (defining two groups); (ii) two Mediterranean macro areas per species (defining four groups); (iii) for *H. polii* populations, two groups divided according to the Mediterranean area (Western HP and Eastern HP); and (iv) for *H. tubulosa* populations, two groups according to the Mediterranean area (Western HT and Eastern HT). For each test the percentage of variance among groups, among populations within groups and within populations have been calculated and the *F* fixation indices *F*_CT_ (differentiation among groups), *F*_SC_ (differentiation within groups among populations) and *F*_ST_ (differentiation within population) have been estimated. The significance test for each AMOVA was calculated setting 1000 random permutations^31^.

## Results

### Inter and intraspecific population genetic structures

A total of 428 sequences of the COI mtDNA gene portion was obtained from 177 to 251 holothurians belonging to *H. tubulosa* and *H. polii*, respectively. After curation of the final alignment, the length of the COI portion was 323 bp. The corresponding assignment of the specimens from which these sequences were obtained was based on the standard morphological criteria described in Methods. Other 13 sequences from additional holothurians, including outgroups, were obtained from four specimens sampled in Croatia coast and nine reference sequences retrieved from GenBank. Overall, from these sequences a total of 51 different COI haplotypes have been identified, for a mean haplotype diversity (*Hd*) of 0.797 (Table 1).

At the species level, *H. polii* and *H. tubulosa* showed a p-distance mean value of 0.180 and a nucleotide diversity mean value of 0.058. The intraspecific p-distance in *H. polii* was lower than the value obtained within *H. tubulosa* (0.009 vs. 0.029 respectively). Similar levels were observed for the π values (0.009 in *H. polii vs.* 0.014 in *H. tubulosa*). The total number of unique haplotypes within *H. polii* and *H. tubulosa* were 33 and 22 with the corresponding *Hd* values of 0.561 and 0.721, respectively (Table 1). These outcomes revealed that *H. polii* may be genetically less diverse than *H. tubulosa*.

The observed overall Tajima’s D and Fu’s Fs values were − 0.3239 (*P* = 0.698) and − 0.1309 (*P =* 0.571) respectively, indicating that the neutral null hypothesis cannot be rejected. In particular, the Tajima’s D statistics for *H. polii* and *H. tubulosa* were − 2.086 and − 2.132 respectively, with significant p-values, that were also obtained for the D statistics calculated for both macro areas (Italian Western and Italian Eastern Mediterranean regions) in both species. On the other hand, the Fu’s Fs test resulted in significant values (*P* < 0.02) only for *H. polii* overall species and *H. polii* Mediterranean macro areas, while *H. tubulosa* resulted with a Fu’s Fs test not significant (Table 1).

Within populations, both Tajima’s D and Fu’s Fs tests resulted in negative values, several statistically significant (Table 1, reported in bold) suggesting recent population expansions in some areas^33^. Moreover, the highest genetic diversity was found in the Ligurian Sea *H. tubulosa* population (p-distance = 0.0239; π = 0.0237), while the lowest genetic diversity was observed in the Sardinian Sea *H. polii* population (p-distance and π = 0.0048). The Ligurian Sea *H. polii* population had the lowest *Hd* diversity (*Hd* = 0.374), while the highest *Hd* was found in the North Adriatic Sea *H. polii* population (*Hd* = 0.877). Table 1 summarises the genetic diversity parameters and the neutrality tests applied within holothurian groups and populations in the investigated Mediterranean areas.

**Table 1:** Inter and intraspecies genetic diversity estimated in *H. Polii* and *H. tubulosa* groups and populations, including *p-distance*, π (nucleotide diversity), *h* (number of haplotypes), *hd* (haplotype diversity) and *Tajima’s D* (statistics of Tajima’s D neutrality test) and associated p-value; *Fu’s fFs* (statistics of Fu’s Fs neutrality test) and associated p-value. HP = *Holothuria polii*; HT = *Holothuria tubulosa*; TM = southern Tyrrhenian Sea; IM = southern Ionian Sea; CS = sicilian Channel Sea; TO = Western Tyrrhenian Sea; AM = southern Adriatic Sea; IS = northern Ionian Sea; TS = northern Tyrrhenian Sea; AS = northern Adriatic Sea; MDS = sardinian sea; ML = Ligurian Sea.

Groups/populations	Num. Seq.	p-distance	π	h	Hd	Tajima’s D^§^	*P* Tajima’s D^§^	Fu’s Fs^§^	*P* Fu’s Fs^§^
Interspecies diversity (*H. polii* vs. *H. tubulosa*)	428	0.1803	0.0580	51*	0.7974*	-0.3239	0.689	-0.1309	0.571
Intraspecies diversity *H. polii*	251	0.0087	0.0085	33	0.5613	**-2.0863**	**0.002**	**-19.0115**	**0.000**
Intraspecies diversity *H. tubulosa*	177	0.0289	0.0135	22	0.7209	**-2.1318**	**0.001**	-5.5394	0.058
Italian Western Mediterranean *H. polii*	129	0.0093	0.0089	27	0.7853	**-1.9910**	**0.003**	**-8.9441**	**0.005**
Italian Eastern Mediterranean *H. polii*	108	0.0087	0.0083	22	0.6387	**-2.5249**	**0.001**	**-6.7208**	**0.012**
Italian Western Mediterranean* H. tubulosa*	105	0.0138	0.0133	19	0.7642	**-2.1382**	**0.001**	-1.6129	0.302
Italian Eastern Mediterranean *H. tubulosa*	72	0.0171	0.0164	23	0.7879	**-2.2915**	**0.002**	-1.7957	0.283
HT_TS	18	0.0066	0.0071	4	0.6994	-0.1097	0.478	**-14.6892**	**0.000**
HT_TO	30	0.0167	0.0151	7	0.7770	**-2.3028**	**0.004**	-5.4037	0.021
HT_TM	26	0.0080	0.0081	6	0.6985	-0.7382	0.249	**-1.8252**	**0.006**
HT_ML	21	0.0239	0.0237	5	0.5952	**-1.9155**	**0.009**	-0.9171	0.327
HT_MDS	10	0.0093	0.0093	6	0.8444	-0.4637	0.321	**-3.7405**	**0.006**
HT_IS	23	0.0136	0.0137	5	0.6403	**-1.9985**	**0.007**	**-6.5284**	**0.001**
HT_AS	24	0.0161	0.0161	6	0.7246	-1.8047	0.017	-1.0472	0.316
HT_AM	25	0.0165	0.0166	9	0.7633	**-2.3462**	**0.002**	-4.2771	0.029
HP_TS	24	0.0188	0.0168	10	0.7826	**-1.6494**	**0.012**	**-24.1498**	**0.000**
HP_TO	29	0.0064	0.0065	7	0.4754	**-1.8767**	**0.012**	**-5.7521**	**0.002**
HP_TM	22	0.0064	0.0065	7	0.6450	-1.2314	0.115	**-15.7000**	**0.000**
HP_ML	34	0.0066	0.0070	8	0.3743	**-1.6987**	**0.016**	**-17.4035**	**0.000**
HP_MDS	20	0.0048	0.0048	5	0.4421	-0.8497	0.225	**-3.3761**	**0.011**
HP_IS	28	0.0062	0.0064	6	0.4815	**-1.6044**	**0.039**	**-19.8258**	**0.000**
HP_IM	33	0.0055	0.0054	8	0.5492	-1.5690	0.172	**-26.7463**	**0.000**
HP_CS	14	0.0065	0.0079	6	0.6044	-1.5623	0.050	**-13.9972**	**0.000**
HP_AS	20	0.0176	0.0187	10	0.8770	**-2.3520**	**0.003**	-3.2490	0.066
HP_AM	27	0.0068	0.0070	9	0.6467	**-1.8481**	**0.016**	**-24.0557**	**0.000**

*The values were obtained including samples of both species.

^§^Significant values of Tajima’s D (*P* < 0.05) and Fu’s Fs (*P* < 0.02) test are reported in bold. The P value of the test is reported in column “*P*”.

Table S1 reports the pairwise p-distance and π values among populations. Here, comparisons among holothurian populations in the investigated Mediterranean sites are represented on a distance matrix, with overall p-distance values ranging from 0.005 to 0.138 and π values ranging from 0.005 to 0.078. Specifically, for populations belonging to the same species, p-distance values ranged from 0.005 to 0.021, while for populations of different species these values ranged from 0.119 to 0.138. Nucleotide diversity showed similar patterns of variation : at an intraspecific level, the minimum π value was 0.005 while the maximum value was 0.020; for population of different species π values ranged from 0.053 to 0.078. Two groups of distance values are clearly highlighted in the heat map of the distance matrix: green boxes represent the minimum values and are within species, while red boxes represent maximum values and are referred to be between different species (Table S1). The most divergent *H. polii* populations were the Northern Adriatic Sea (AS) and Northern Tyrrhenian Sea (TS) populations (p-distance = 0.018; π = 0.018). The most divergent *H. tubulosa* populations were the Northern Adriatic Sea (AS) and Ligurian Sea (ML) populations (p-distance = 0.02; π = 0.02), the Ligurian Sea (ML) and Western Tyrrhenian Sea (TO) populations (p-distance = 0.019; π = 0.021) and the Southern Adriatic Sea (AM) and Ligurian Sea (ML) populations (p-distance = 0.019; π = 0.021).

The genetic differentiation among populations was also estimated with the fixation index F_ST_, Gst and Gammast parameters (Table S2). Within species, the highest F_ST_ value obtained within *H. polii*, the highest F_ST_value was between the Northern and the Western Tyrrhenian Sea populations (F_ST_ = 0.1147, *P* = 0.000), while the highest Gst and Gammast values were for the Northern Tyrrhenian Sea and Ligurian Sea (Gst = 0.0474; Gammast = 0.0855). Within *H. tubulosa* the highest F_ST_ and Gammast values were observed for the Sardinian Sea and North-Eastern Ionian Sea populations (F_ST_ = 0.0685, *P* = 0.295; Gammast = 0.0603) while the highest Gst value was for the Sardinian Sea and Ligurian Sea populations (Gst = 0.0880). Considering that generally any value of Gammast index lower than 0.15 indicate a lack of differentiation^36,37^, these results may indicate an active gene flow within species. Among species, the highest F_ST_ and Gammast values were observed for the *H. tubulosa* Northern Tyrrhenian Sea populations and the *H. polii* Sardinian Sea populations (F_ST_ = 0.9447, *P* = 0.000; Gammast = 0.9019). The highest Gst value was obtained between *H. tubolosa* and *H. polii* Ligurian Sea populations (Gst = 0.3470). Finally, considering the populations of the two main Mediterranean macro areas (Italian Western and Eastern Mediterranean areas), for *H. tubulosa* F_ST_, Gammast and Gst values were 0.0041 (*P* = 0.325), 0.0038 and − 0.0007 respectively; whereas for *H. polii* F_ST_, Gammast and Gst values were 0.0002 (*P* = 0.584), 0.0041 and − 0.0005 respectively (Table S2).

AMOVA results for the four genetic structures tested are reported in Table 2. All AMOVA analyses revealed that most of molecular variance is distributed within populations (91.49%, 92.10%, 95.78% and 91.02% for the four analyses respectively), while the remaining genetic diversity was distributed within groups among populations and the least variance among groups (Table 2). All fixation indices, including *F*_CT_, *F*_SC_ and *F*_ST,_ were less than 0.1 and most of them resulted statistically significant (*P* < 0.05): only the *F*_CT_ indices in both *H. polii* and *H*,* tubulosa* specific AMOVA analyses were not significant. The overall AMOVA results indicate an absence of genetic structure in *H. polii* and *H. tubulosa* Mediterranean populations.

**Table 2:** Analysis of molecular variance (AMOVA) in *H. Polii* and *H. tubulosa* populations. D.f. = degrees of freedom; *F*_CT_: index of differentiation among groups; *F*_SC_: index of differentiation within groups among populations; *F*_ST_: index of differentiation within populations. Significant p-values are in bold.

Structures	d.f.	Sum of squares	Variance components	% of variation	Fixation indices	*P*
*2 groups (H. polii; H. tubulosa)*						
Among groups	1	3.735	Va: 0.01215	2.43%	*F*_CT_: 0.02430	**0.000**
Among populations within groups	16	18.818	Vb: 0.03041	6.08%	*F*_SC_: 0.06231	**0.000**
Within populations	410	187.669	Vc: 0.45773	91.49%	*F*_ST_: 0.08509	**0.000**
Total	427	210.222	0.50030	100.00%		
*4 groups (Western HP; Eastern HP; Western HT; Eastern HT)*						
Among groups	3	5.869	Va: 0.00658	1.33%	*F*_CT_: 0.01328	**0.034**
Among populations within groups	13	16.105	Vb: 0.03258	6.57%	*F*_SC_: 0.06662	**0.000**
Within populations	397	181.240	Vc: 0.45653	92.10%	*F*_ST_: 0.07901	**0.000**
Total	413	203.215	0.49569	100.00%		
*H. polii –* 2 groups (*Western HP; Eastern HP)*						
Among groups	1	0.923	Va: -0.00120	-0.25%	*F*_CT_: -0.00247	0.515
Among populations within groups	7	7.259	Vb: 0.02184	4.47%	*F*_SC_: 0.04460	**0.000**
Within populations	228	106.662	Vc: 0.46782	95.78%	*F*_ST_: 0.04225	**0.000**
Total	236	114.844	0.48845	100.00%		
*H. tubulosa –* 2 groups (*Western HP; Eastern HP)*						
Among groups	1	1.213	Va: -0.00422	-0.87%	*F*_CT_: -0.00871	0.629
Among populations within groups	6	8.847	Vb: 0.04778	9.86%	*F*_SC_: 0.09770	**0.000**
Within populations	169	74.578	Vc: 0.44129	91.02%	*F*_ST_: 0.08984	**0.000**
Total	176	84.638	0.48485	100.00%		

### Phylogenetic relationships between the obtained mtDNA sequences

**Fig. 2:**
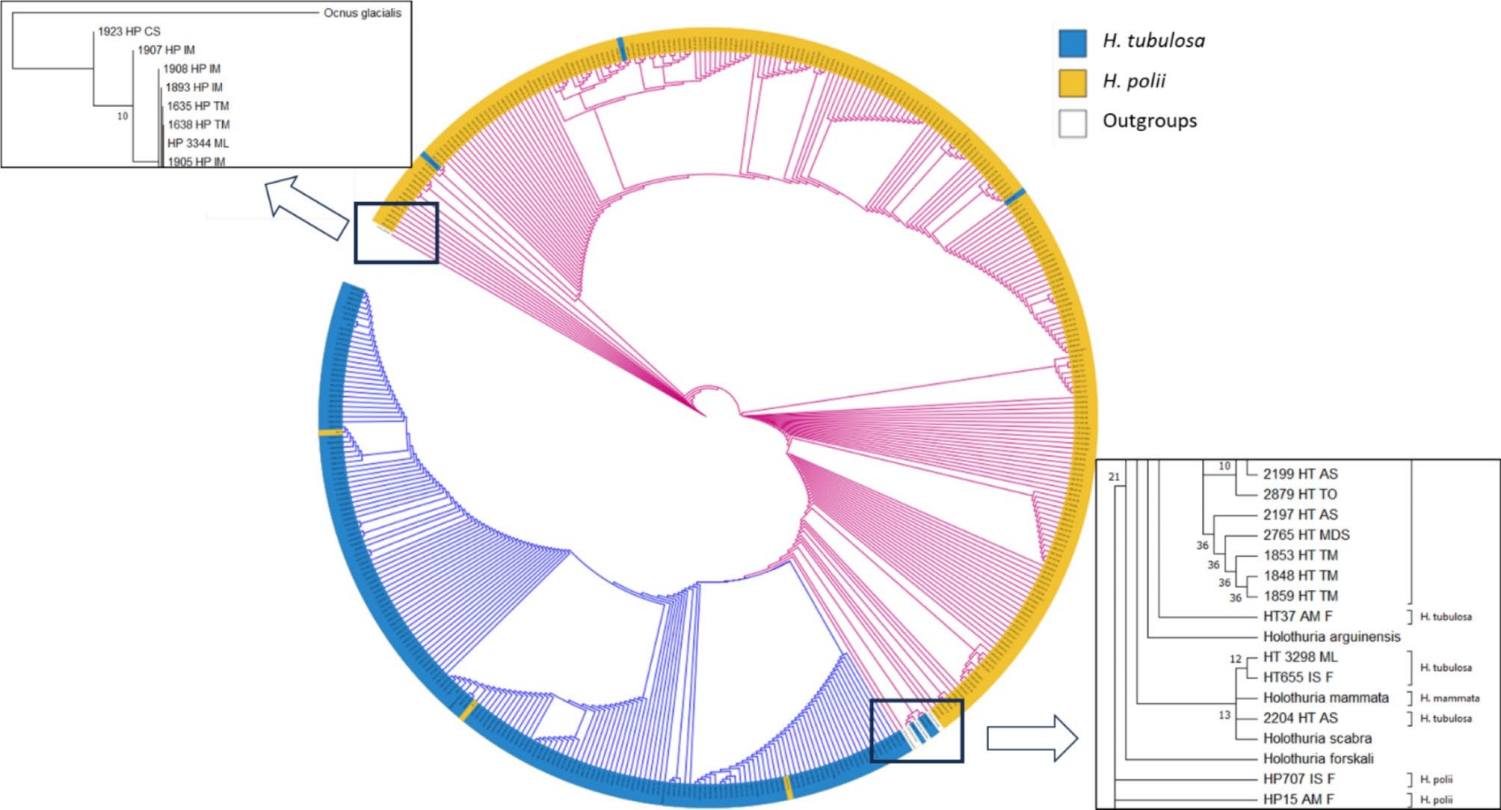
Circular Neighbour-Joining (NJ) phylogenetic tree based on the obtained *H. polii* and *H. tubulosa* COI mtDNA sequences. The NJ tree highlights the *H. tubulosa* clade in blue and the *H. polii* clade in pink and yellow. The boxes detailed the phylogenetic positions of the outgroups.

Phylogenetic analysis was based on the obtained COI mtDNA sequences (Fig. 2). The Neighbour-Joining phylogenetic tree clearly showed the presence of two major clades each assigned to one of the two species, *H. polii* and *H. tubulosa*, with a few peculiar features and exceptions. The outgroup *O. glacialis* was placed at the base of the *Holothuria* clades. The *H. polii* clade was positioned at the base in relation to the *H. tubulosa* clade, which forms a monophyletic cluster with a statistically supported node (node bootstrap value of 86). Other *Holothuria* species are also positioned within these two major clades: in particular, *H. forskali* and *H. scabra* were located within the *H. polii* clade in a basal position relative to the *H. tubulosa* clade, while *H. arguinensis* and *H. mammata* taxa were located within the *H. tubulosa* clade (Fig. 2). It was interesting to note that *H. mammata* formed a cluster with three holothurians attributed to *H. tubulosa*, positioned at the base in relation to the other sequences from *H. tubulosa* samples. Sequences from these three specimens had an average p-distance with *H. mammata* and *H. tubulosa* of 0.003 and 0.102 respectively, suggesting that they may likely belong to the *H. mammata* species. Surprisingly, a few sequences obtained from some specimens morphologically attributed to one species (*H. polii* or *H. tubulosa*) were positioned within the other species cluster. For example, sequences from three specimens attributed to *H. polii* (sample HP2232_AS from Northern Adriatic Sea, sample HP959_TS from Northern Tyrrhenian Sea and sample HP2 from Croatia) clustered together within the *H. tubulosa* cluster and sequences obtained from three specimens assigned to *H. tubulosa* species (sample HT50_AM from Southern Adriatic Sea, sample HT2910_TO from Western Tyrrhenian Sea and sample HT3313_ML from Ligurian Sea) clustered in the *H. polii* clade. In particular, the mean p-distance value between the three HP samples that clustered within *H. tubulosa* clade with the morphological corresponding species (*H. polii*) was 0.198 while the mean p-distance with *H. tubulosa* species was 0.008. On the other hand, the three HT samples that clustered in the *H. polii* clade with the morphological corresponding species (*H. tubulosa*) was 0.198 while the mean p-distance value with *H. polii* species was 0.007. These results indicated that these specimens genetically belong to the other *Holothuria* species, despite their morphological assignment. Furthermore, phylogenetic analysis revealed that there was no clear topological structure in the NJ tree among Mediterranean areas, as sequences derived the same holothurian species (*H. polii* or *H. tubulosa*) but from different populations clustered together regardless of their geographical origin. Therefore, there were no phylogenetic patterns that could be explained by any geographic gradients.

## Discussion

In this study we analysed, for the first time, the genetic diversity of several holothurian populations belonging to two species (*H. polii* and *H. tubulosa*) distributed in ten Mediterranean areas through the Italian coasts.

The results strongly indicate that these two sympatric species are genetically different, with a genetic p-distance of 0.18 and a π value of 0.078 for the COI mtDNA gene. These values are higher than the average thresholds that are usually set to define different species, which are 0.03 for p-distance and 0.02 for nucleotide diversity π for the COI mtDNA gene^38,39^. In the Metazoan clade, the separation between two species based on COI mtDNA derived genetic distance (considering both p-distance and π) includes values ranging from 0.02 to 0.67, with maximum values in Mollusca and Arthropoda phyla^40^. Therefore, in this study the genetic distance between *H. polii* and *H. tubulosa* was in agreement with what may be expected in Metazoan invertebrates, confirming that the COI mtDNA gene is a good species-specific marker also in holothurians.

Considering intraspecies genetic diversity, *H. polii* vs. *H. tubulosa* values were below the threshold as expected in the invertebrate Metazoan clade. In fact, the intraspecific p-distance and nucleotide diversity value can reach 0.68 in some mollusks and arthropods due to peculiar mitochondrial evolution^40^, but generally is below 0.03^38^. Interestingly, *H. polii* showed a lower genetic distance than *H. tubulosa* and the difference between intraspecific genetic distance within these two species could be due to the different survival strategy of the planktonic larvae before they begin the benthonic life and settle on the seabed^2,11^. In fact, even if the larval development period of *H. polii* is generally shorter than *H. tubulosa*^41^, the offspring of *H. polii* is considerably larger than the other congeneric Mediterranean species including *H. tubulosa* (average diameter 203.7 ± 10.21 μm vs. 151.2 ± 1.7 μm, respectively^2,11^). This difference in size reflects in a reproductive strategy of *H. polii* with higher larvae survivorship, since larvae can survive even without feeding, showing a facultative planktotrophic feeding behavior in this species, thanks to the support of a larger yolk^14^. This reproductive strategy probably supports a major dispersion in *H. polii*, by resulting in a more genetic homogeneity and a lower genetic diversity compared to *H. tubulosa*^14^. Nevertheless, this is only hypothetical since other factors such as fecundity and larval behavior (i.e., tolerance to environmental gradients), as well as the response of both species to palaeogeographical and paleoclimatic shifts within the Mediterranean Basin (i.e. during the Pleistocene glacial and interglacial cycles), could also be abetting on the emerging patterns.

The results obtained in this study showed that different holothurian populations are genetically homogeneous within species (Table 1) and this suggests an active gene flow in all Mediterranean areas investigated. This is also supported by the AMOVA analysis that revealed that most of the genetic variability (> 91%) was within populations, indicating an absence of genetic structure within *H. polii* and *H. tubulosa* species. Moreover, fixation indices obtained from AMOVA resulted less than 0.1 (Table 2), as well as the F_ST_ index obtained from intraspecies population pairwise comparisons (Table S2). Indeed, F_ST_ index is usually estimated in order to evaluate genetic differentiation among populations^42^ and our results showed intraspecific low F_ST_ values (maximum F_ST_ was detected between two *H. polii* Thyrrenian populations: F_ST_ = 0.1147, *P* = 0.000) indicating a high genetic exchange and no differentiation among the different geographical populations belonging to the same species^43,44^. An explanation could be that the planktonic larval stage of these species can last from days to weeks and the natant larvae can reach considerable distances carried by marine currents. This probably guarantees a continuous gene flow among sea cucumber populations preventing their genetic diversification^2,11^. Indeed, the genetic variability of a marine species could be shaped by marine barriers such as narrow passages between land masses, types of currents, salinity gradients, or other anthropogenic barriers^42^. For example, Vergara-Chen et al.^17^ detected high levels of haplotype diversity and low values of nucleotide diversity at micro-geographic scale among *H. polii* populations across Mar Menor coastal lagoon and nearby marine areas. The Mediterranean Sea can present such barriers also at macro-geographic scale: for example, a potential gene flow barrier could be the Siculo-Tunisian Strait which separates the Eastern from the Western Mediterranean Basin^45^. However, according to Borrero-Pérez et al.^4^, who studied the genetic structure of the sea cucumber *H. mammata* populations across the Northeast Atlantic Ocean and Mediterranean Sea by analysing the mitochondrial COI and 16S genes, *H. mammata* populations from the Macaronesian islands (Atlantic Ocean) and the West Mediterranean could be considered a panmictic metapopulation. Our results agree with this hypothesis, since the F_ST_ values obtained from both *H. polii* and *H. tubulosa* populations from Western and Eastern Mediterranean macro areas suggest no genetic structures and a complete gene flow among different populations. This suggests that the Siculo-Tunisian Strait is not a barrier to gene flow for the holothurian planktonic larval stage even for *H. polii* and *H. tubulosa* species, also in agreement to Borrero-Pérez et al.^4^. However, other studies conducted on a wider geographical scale with a limited number of populations, such as the investigation of Valente et al.^16^ highlighted a slight genetic differentiation (COI gene), but still significant, between the Western and Eastern Mediterranean populations of *H. polii*, with a higher genetic diversity in the East. Based on these results, the authors suggested that this region could be the origin of the subsequent colonization through the Mediterranean Sea^16,22^. Therefore, further investigations using longer gene fragments will be helpful to better understand the genetic structure on the Mediterranean scale.

The Neighbour-Joining phylogenetic tree obtained in this study showed two major clades corresponding to the *H. tubulosa* and *H. polii* species. As expected, the *H. polii* clade appeared at the base of the tree in relation to the *H. tubulosa* clade, supporting the previously identified phylogenetic relationship between the two species^46–51^. This relationship was also reported by other authors who analysed the mitochondrial genes 16S and COI^46–51^.However, sequences obtained from some individual specimens were unexpectedly placed in the tree in contrast to their morphological species assignment. For example, sequences from three samples resembling *H. tubulosa* clustered within the *H. mammata* sequence cluster, with a p-distance very similar to *H. mammata* rather than *H. tubulosa*. In these cases, it is highly likely that these three samples actually belong to the *H. mammata* species but exhibit a morphological phenotype very similar to *H. tubulosa* due to morphological overlap within the two species. There is a known morphological overlap among certain *Holothuria* species such as *H. tubulosa*, *H. mammata*, *H. arguinensis* and *H. stellati*, highlighting the necessity of an integrative taxonomical approach that combines morphology and genetic information for accurate sea cucumber species identification^50^. Cryptic species are common within the Holothuriidae family, making it challenging to morphologically distinguish *H. mammata* and *H. tubulosa*^51^. Both species exhibit significant intraspecies morphological variation, with numerous morphotypes that overlap between the two species, often misidentified as *H. tubulosa* but genetically distinct from it^51,52^. Additionally, the position of *H. arguinensis* within the *H. tubulosa* clade in the NJ phylogenetic tree confirmed the close genetic relationship between these species, which are frequently confused morphologically, as noted by previous studies^14,52,53^.

Another interesting result concerned the unexpected position in the phylogenetic tree of three *H. polii*-like samples which clustered in the *H. tubulosa* clade and three *H. tubulosa*-like specimens, which were in the *H. polii* cluster. In these cases, there is no morphological overlap to explain this discrepancy between morphology and genetics, as these two species are phenotypically distinguishable^2,11,14^. On the contrary, this phenomenon could be due to hybridization events that may have occurred between these two sympatric species. Indeed, hybridization implies an exchange of gametes depending on the level of gene flow and a recent study indicated that it is possible between *H. polii* and *H. tubulosa* in the Eastern Mediterranean Sea^54^. Since mitochondrial genes are maternally inherited and it is difficult to detect hybridization and introgression events by analysing only mtDNA, further analyses including nuclear markers are needed to determine the presence of hybrids. Nonetheless, the results suggest that two-way hybridization events may have occurred between the two species, confirming what has already been reported by Gkafas et al.^54^. This phenomenon could be related to an introgressive adaptation to a changing environment, in which *H. polii* and *H. tubulosa* may take advantage in the natural selection process^55,56^.

Finally, the Tajima’s and Fu’s neutrality tests computed on the entire dataset failed to reject the null hypothesis of neutral evolution in the COI mtDNA gene (Tajima’s D statistic = -0.3239, *P* = 0.698; Fu’s Fs statistic = -0.1309, *P* = 0.517), indicating that, generally for this locus, holothurians populations have not been subjected to selection pressure. However, when considering species and populations separately, Tajima’s D and Fu’s Fs always showed negative values, in several cases statistically significant (Table 1). The tests of neutrality with significant negative Tajima’s D and Fu’s Fs values may suggest population expansions after a bottleneck or a selective sweep with an excess of low frequency variations^33,34,56,57^. Moreover, considering the overall low nucleotide diversity (π) and the relatively high haplotype diversity (*Hd*) parameters obtained from all population of both species, according to Grant and Bowen^58^ this situation can be associated with a recent demographic population expansion after a bottleneck^58^. This is in agreement with what we obtained from the neutrality tests. Indeed, for both species, the results may indicate that some haplotypes have selective advantages over others, leading to an excess of low frequency polymorphisms relative to the expectation. The bottleneck that caused the recent expansion could be due to several factors related to these holothurian population dynamics, including mass mortality events from new pathogens or environmental changes in the Mediterranean basin^59,60^.

In conclusion, this study provides an overview of the genetic variability in several populations of *H. polii* and *H. tubulosa* in ten Mediterranean locations using a 323 bp portion of the COI gene. The results suggest an active gene flow among populations for both species in all areas investigated as well as a recent population expansion of both species after a bottleneck, with promising perspective on their conservation strategy. Further analyses with longer and more informative mtDNA regions as well as using nuclear DNA polymorphisms are needed to confirm the presence of hybridization events suggested by the results. These genetic data will be potentially useful in establishing farmed stocks with a high level of genetic variability for aquaculture aimed at both human consumption and restocking. On the other hand, implementing management and conservation actions for these two holothurian species will also benefit from the genetic information obtained for the investigated Mediterranean populations.

## Electronic supplementary material

Below is the link to the electronic supplementary material.

Supplementary Material 1

## Data Availability

All dataset and sequences have been deposited in ENA Archive with the project number PRJEB77360.
