# Supplementary material for: Monitoring the genetic diversity in holothurian populations from the Italian coasts with mitochondrial DNA sequences
Source: Sci Rep. 2024 Oct 19;14:24592. doi: 10.1038/s41598-024-76087-5 (PMC11490579; doi:10.1038/s41598-024-76087-5)
Supplement: Supplementary file 1 — Supplementary Material 1 [file 41598_2024_76087_MOESM1_ESM.docx]

**Monitoring the genetic diversity in holothurian populations from the Italian coasts with mitochondrial DNA sequences**

Anisa Ribani, Valeria Taurisano, Arnold Rakaj, Alessandra Fianchini, Luca Grosso, Davide Pensa, Domitilla Pulcini, Luca Buttazzoni, Giuseppina Schiavo, Samuele Bovo, Francesca Bertolini, Valerio Joe Utzeri, Fausto Tinti, Fabrizio Capoccioni, Luca Fontanesi

**Supplementary material**

**Table S1.** Heatmap of genetic diversity between populations. The part of the matrix below the diagonal reports the p-distance, while the part above the diagonal reports the nucleotide diversity π (in bold and italics). Variation of colours indicate the range of p-distance and π parameter values. Green: lowest values; Red: highest values. HP = *Holothuria polii*; HT = *Holothuria tubulosa*; TM = Southern Tyrrhenian Sea; IM = Southern Ionian Sea; CS = Sicilian Channel Sea; TO = Western Tyrrhenian Sea; AM = Southern Adriatic Sea; IS = Northern Ionian Sea; TS = Northern Tyrrhenian Sea; AS = Northern Adriatic Sea; MDS = Sardinian Sea; ML = Ligurian Sea

| *Population* | *HP-*  *TS* | | *HP-ML* | *HP-*  *IS* | | | *HP-AM* | | *HP-*  *TO* | *HP-*  *AS* | | *HP-*  *MS* | *HP-*  *IM* | *HP-TM* | *HP-*  *CS* | | *HT-*  *AS* | | *HT-ML* | *HT-*  *MS* | | *HT-*  *TS* | | *HT-*  *IS* | | *HT-*  *AM* | | *HT-*  *TO* | | *HT-TM* |
| --- | --- | --- | --- | --- | --- | --- | --- | --- | --- | --- | --- | --- | --- | --- | --- | --- | --- | --- | --- | --- | --- | --- | --- | --- | --- | --- | --- | --- | --- | --- |
| *HP_TS* |  | ***0.012*** | | | ***0.012*** | ***0.013*** | | ***0.012*** | | ***0.018*** | ***0.012*** | | ***0.012*** | ***0.014*** | | ***0.016*** | | ***0.06*** | ***0.058*** | | ***0.053*** | | ***0.058*** | ***0.06*** | ***0.059*** | | ***0.058*** | | ***0.058*** | |
| *HP_ML* | 0.014 |  | | | ***0.006*** | ***0.007*** | | ***0.007*** | | ***0.012*** | ***0.006*** | | ***0.006*** | ***0.007*** | | ***0.008*** | | ***0.076*** | ***0.068*** | | ***0.057*** | | ***0.064*** | ***0.070*** | ***0.069*** | | ***0.07*** | | ***0.077*** | |
| *HP_IS* | 0.013 | 0.006 | | |  | ***0.007*** | | ***0.006*** | | ***0.011*** | ***0.005*** | | ***0.005*** | ***0.007*** | | ***0.008*** | | ***0.071*** | ***0.070*** | | ***0.056*** | | ***0.067*** | ***0.072*** | ***0.070*** | | ***0.072*** | | ***0.072*** | |
| *HP_AM* | 0.014 | 0.007 | | | 0.006 |  | | ***0.007*** | | ***0.012*** | ***0.006*** | | ***0.005*** | ***0.007*** | | ***0.008*** | | ***0.072*** | ***0.070*** | | ***0.058*** | | ***0.068*** | ***0.073*** | ***0.071*** | | ***0.072*** | | ***0.072*** | |
| *HP_TO* | 0.013 | 0.006 | | | 0.006 | 0.007 | |  | | ***0.011*** | ***0.006*** | | ***0.006*** | ***0.007*** | | ***0.008*** | | ***0.074*** | ***0.071*** | | ***0.058*** | | ***0.066*** | ***0.072*** | ***0.07*** | | ***0.071*** | | ***0.075*** | |
| *HP_AS* | 0.018 | 0.012 | | | 0.012 | 0.012 | | 0.012 | |  | ***0.012*** | | ***0.011*** | ***0.012*** | | ***0.015*** | | ***0.075*** | ***0.075*** | | ***0.070*** | | ***0.070*** | ***0.073*** | ***0.071*** | | ***0.069*** | | ***0.074*** | |
| *HP_MS* | 0.012 | 0.006 | | | 0.005 | 0.006 | | 0.006 | | 0.011 |  | | ***0.005*** | ***0.006*** | | ***0.007*** | | ***0.074*** | ***0.074*** | | ***0.066*** | | ***0.069*** | ***0.073*** | ***0.070*** | | ***0.069*** | | ***0.076*** | |
| *HP_IM* | 0.013 | 0.006 | | | 0.006 | 0.006 | | 0.006 | | 0.012 | 0.005 | |  | ***0.006*** | | ***0.006*** | | ***0.077*** | ***0.073*** | | ***0.058*** | | ***0.067*** | ***0.075*** | ***0.073*** | | ***0.073*** | | ***0.078*** | |
| *HP_TM* | 0.014 | 0.006 | | | 0.007 | 0.007 | | 0.006 | | 0.012 | 0.006 | | 0.006 |  | | ***0.008*** | | ***0.076*** | ***0.074*** | | ***0.066*** | | ***0.069*** | ***0.073*** | ***0.071*** | | ***0.07*** | | ***0.076*** | |
| *HP_CS* | 0.014 | 0.006 | | | 0.006 | 0.007 | | 0.006 | | 0.012 | 0.006 | | 0.006 | 0.006 | |  | | ***0.074*** | ***0.074*** | | ***0.075*** | | ***0.069*** | ***0.071*** | ***0.068*** | | ***0.064*** | | ***0.07*** | |
| *HT_AS* | 0.123 | 0.136 | | | 0.130 | 0.135 | | 0.136 | | 0.130 | 0.135 | | 0.136 | 0.135 | | 0.136 | |  | ***0.02*** | | ***0.014*** | | ***0.011*** | ***0.014*** | ***0.015*** | | ***0.015*** | | ***0.012*** | |
| *HT_ML* | 0.119 | 0.127 | | | 0.125 | 0.13 | | 0.13 | | 0.126 | 0.129 | | 0.129 | 0.13 | | 0.129 | | 0.02 |  | | ***0.019*** | | ***0.016*** | ***0.019*** | ***0.02*** | | ***0.019*** | | ***0.014*** | |
| *HT_MS* | 0.124 | 0.137 | | | 0.131 | 0.136 | | 0.137 | | 0.132 | 0.136 | | 0.137 | 0.137 | | 0.137 | | 0.013 | 0.017 | |  | | ***0.008*** | ***0.012*** | ***0.014*** | | ***0.013*** | | ***0.009*** | |
| *HT_TS* | 0.123 | 0.132 | | | 0.131 | 0.132 | | 0.132 | | 0.127 | 0.132 | | 0.132 | 0.132 | | 0.131 | | 0.010 | 0.015 | | 0.007 | |  | ***0.011*** | ***0.013*** | | ***0.012*** | | ***0.007*** | |
| *HT_IS* | 0.124 | 0.131 | | | 0.131 | 0.132 | | 0.132 | | 0.127 | 0.131 | | 0.132 | 0.131 | | 0.131 | | 0.013 | 0.017 | | 0.011 | | 0.01 |  | ***0.015*** | | ***0.015*** | | ***0.010*** | |
| *HT_AM* | 0.119 | 0.126 | | | 0.126 | 0.127 | | 0.127 | | 0.122 | 0.126 | | 0.127 | 0.126 | | 0.126 | | 0.015 | 0.019 | | 0.012 | | 0.011 | 0.015 |  | | ***0.016*** | | ***0.011*** | |
| *HT_TO* | 0.12 | 0.133 | | | 0.127 | 0.132 | | 0.133 | | 0.128 | 0.132 | | 0.133 | 0.133 | | 0.133 | | 0.016 | 0.021 | | 0.013 | | 0.011 | 0.014 | 0.016 | |  | | ***0.011*** | |
| *HT_TM* | 0.124 | 0.138 | | | 0.132 | 0.137 | | 0.138 | | 0.132 | 0.137 | | 0.138 | 0.138 | | 0.138 | | 0.012 | 0.016 | | 0.009 | | 0.006 | 0.010 | 0.011 | | 0.012 | |  | |

**Table S2.** Holothurian population structure indices. Hs: weighted average of estimated haplotype diversities in the subpopulations, Ks: number of synonymous substitutions per synonymous site, Kxy: average number of nucleotide differences between populations, Gst: measure of population differentiation, Gammast: gamma statistics, F_st_: fixation index; F_st_ *P*: p-value associated to F_st_. HP = *Holothuria polii*; HT = *Holothuria tubulosa*; TM = Southern Tyrrhenian Sea; IM = Southern Ionian Sea; CS = Sicilian Channel Sea; TO = Western Tyrrhenian Sea; AM = Southern Adriatic Sea; IS = Northern Ionian Sea; TS = Northern Tyrrhenian Sea; AS = Northern Adriatic Sea; MDS = Sardinian Sea; ML = Ligurian Sea.

In bold: statistically significant

| Population 1 | Population 2 | Hs | Ks | Kxy | Gst | Gammast | F_st_ | *P* F_st_ |
| --- | --- | --- | --- | --- | --- | --- | --- | --- |
| HT_TS | HT_TO | 0.748770 | 1.57902 | 1.45185 | -0.00869 | 0.01476 | -0.00783 | 0.81455 |
| HT_TS | HT_TM | 0.698820 | 1.04182 | 1.00427 | -0.01130 | 0.00832 | -0.03098 | 0.83887 |
| HT_TS | HT_ML | 0.642830 | 1.76410 | 1.70635 | 0.01091 | 0.02379 | -0.00186 | 0.58594 |
| HT_TS | HT_MDS | 0.747710 | 1.15079 | 1.21111 | 0.01338 | 0.04092 | 0.00001 | 0.42871 |
| HT_TS | HT_IS | 0.665840 | 1.28603 | 1.23430 | -0.00019 | 0.01582 | -0.01672 | 0.79004 |
| HT_TS | HT_AS | 0.713990 | 1.32505 | 1.25463 | 0.00432 | 0.01175 | -0.02374 | 0.73633 |
| HT_TS | HT_AM | 0.737080 | 1.68217 | 1.56222 | -0.01290 | 0.01397 | -0.01565 | 0.95801 |
| HT_TS | HP_TS | 0.747550 | 1.77847 | 12.38889 | 0.13885 | 0.75670 | **0.86430** | **0.00000** |
| HT_TS | HP_TO | 0.558710 | 0.83891 | 13.65326 | 0.25858 | 0.88315 | **0.93632** | **0.00000** |
| HT_TS | HP_TM | 0.669170 | 1.05238 | 13.58586 | 0.19526 | 0.86174 | **0.92289** | **0.00000** |
| HT_TS | HP_ML | 0.482670 | 0.76224 | 13.69281 | 0.30200 | 0.88886 | **0.94025** | **0.00000** |
| HT_TS | HP_MDS | 0.563160 | 0.73130 | 13.47222 | 0.27516 | 0.90194 | **0.94472** | **0.00000** |
| HT_TS | HP_IS | 0.564480 | 0.76329 | 13.54365 | 0.25670 | 0.89313 | **0.94052** | **0.00000** |
| HT_TS | HP_IM | 0.600340 | 0.80515 | 13.63131 | 0.22288 | 0.88354 | **0.93769** | **0.00000** |
| HT_TS | HP_CS | 0.658650 | 0.92788 | 13.79365 | 0.20696 | 0.87970 | **0.93348** | **0.00000** |
| HT_TS | HP_AS | 0.702480 | 1.65651 | 13.25000 | 0.17045 | 0.78798 | **0.87746** | **0.00000** |
| HT_TS | HP_AM | 0.678510 | 0.98545 | 13.76068 | 0.18519 | 0.86824 | **0.92950** | **0.00000** |
| HT_TO | HT_TM | 0.740760 | 1.52916 | 1.52051 | 0.00401 | 0.02453 | 0.01441 | 0.27637 |
| HT_TO | HT_ML | 0.703530 | 2.12928 | 2.21111 | 0.03926 | 0.02946 | 0.01735 | 0.47559 |
| HT_TO | HT_MDS | 0.792000 | 1.80038 | 1.63333 | 0.00169 | 0.01341 | -0.02510 | 0.78613 |
| HT_TO | HT_IS | 0.718430 | 1.74567 | 1.77971 | 0.02360 | 0.03565 | 0.03458 | 0.27539 |
| HT_TO | HT_AS | 0.753970 | 1.76751 | 1.76250 | 0.01050 | 0.02258 | 0.00843 | 0.51367 |
| HT_TO | HT_AM | 0.770840 | 2.03866 | 2.03067 | -0.00125 | 0.01403 | -0.00946 | 0.6123 |
| HT_TO | HP_TS | 0.779470 | 2.12016 | 11.95972 | 0.10814 | 0.70544 | **0.82070** | **0.00000** |
| HT_TO | HP_TO | 0.628930 | 1.34274 | 13.14138 | 0.21752 | 0.82035 | **0.89859** | **0.00000** |
| HT_TO | HP_TM | 0.722020 | 1.57478 | 13.07576 | 0.15451 | 0.78906 | **0.88446** | **0.00000** |
| HT_TO | HP_ML | 0.562250 | 1.24109 | 13.18235 | 0.26034 | 0.83200 | **0.90279** | **0.00000** |
| HT_TO | HP_MDS | 0.645960 | 1.35165 | 12.96667 | 0.21483 | 0.81298 | **0.90684** | **0.00000** |
| HT_TO | HP_IS | 0.634720 | 1.29145 | 13.03333 | 0.21455 | 0.82536 | **0.90265** | **0.00000** |
| HT_TO | HP_IM | 0.657340 | 1.28342 | 13.11515 | 0.19270 | 0.82635 | **0.89991** | **0.00000** |
| HT_TO | HP_CS | 0.725230 | 1.57921 | 13.28333 | 0.14997 | 0.77392 | **0.89605** | **0.00000** |
| HT_TO | HP_AS | 0.748940 | 2.05481 | 12.78167 | 0.12945 | 0.72418 | **0.83673** | **0.00000** |
| HT_TO | HP_AM | 0.725140 | 1.48488 | 13.25385 | 0.15146 | 0.80449 | **0.89184** | **0.00000** |
| HT_TM | HT_ML | 0.652850 | 1.67319 | 1.71978 | -0.00840 | 0.01614 | -0.01461 | 0.3291 |
| HT_TM | HT_MDS | 0.734960 | 1.16840 | 1.30769 | 0.03951 | 0.05286 | 0.0468 | 0.20508 |
| HT_TM | HT_IS | 0.671330 | 1.27688 | 1.26421 | -0.01414 | 0.01099 | -0.02065 | 0.52441 |
| HT_TM | HT_AS | 0.710980 | 1.30984 | 1.30449 | -0.00563 | 0.01485 | -0.01174 | 0.28906 |
| HT_TM | HT_AM | 0.730210 | 1.61124 | 1.59539 | -0.00965 | 0.01193 | -0.01671 | 0.87695 |
| HT_TM | HP_TS | 0.738710 | 1.69071 | 12.40865 | 0.14524 | 0.76809 | **0.86167** | **0.00000** |
| HT_TM | HP_TO | 0.580350 | 0.89579 | 13.66048 | 0.26177 | 0.88079 | **0.93376** | **0.00000** |
| HT_TM | HP_TM | 0.674170 | 1.08198 | 13.60489 | 0.19483 | 0.85751 | **0.9204** | **0.00000** |
| HT_TM | HP_ML | 0.513240 | 0.82461 | 13.70588 | 0.30680 | 0.88822 | **0.93772** | **0.00000** |
| HT_TM | HP_MDS | 0.588590 | 0.81803 | 13.48462 | 0.26480 | 0.88869 | **0.94215** | **0.00000** |
| HT_TM | HP_IS | 0.585630 | 0.83243 | 13.55357 | 0.25894 | 0.88816 | **0.93795** | **0.00000** |
| HT_TM | HP_IM | 0.614360 | 0.86275 | 13.64569 | 0.23180 | 0.88335 | **0.93516** | **0.00000** |
| HT_TM | HP_CS | 0.667110 | 0.98831 | 13.80220 | 0.19405 | 0.86187 | **0.93096** | **0.00000** |
| HT_TM | HP_AS | 0.701380 | 1.58233 | 13.26731 | 0.16843 | 0.79179 | **0.87495** | **0.00000** |
| HT_TM | HP_AM | 0.681540 | 1.02308 | 13.77219 | 0.18939 | 0.86667 | **0.92698** | **0.00000** |
| HT_ML | HT_MDS | 0.669080 | 2.09749 | 2.04762 | 0.08804 | 0.05359 | 0.06202 | 0.19922 |
| HT_ML | HT_IS | 0.618900 | 1.94380 | 1.91097 | -0.01760 | 0.00984 | -0.02799 | 0.8877 |
| HT_ML | HT_AS | 0.664670 | 1.96560 | 1.97024 | 0.00364 | 0.01735 | -0.01203 | 0.61719 |
| HT_ML | HT_AM | 0.687290 | 2.28551 | 2.24000 | 0.00751 | 0.01008 | -0.02509 | 0.72168 |
| HT_ML | HP_TS | 0.695780 | 2.38879 | 12.04564 | 0.18033 | 0.68039 | **0.80153** | **0.00000** |
| HT_ML | HP_TO | 0.524880 | 1.44457 | 13.20033 | 0.30127 | 0.80482 | **0.88038** | **0.00000** |
| HT_ML | HP_TM | 0.620770 | 1.74175 | 13.13636 | 0.23421 | 0.77530 | **0.86624** | **0.00000** |
| HT_ML | HP_ML | 0.456630 | 1.31703 | 13.22129 | 0.34697 | 0.81485 | **0.88445** | **0.00000** |
| HT_ML | HP_MDS | 0.520740 | 1.47779 | 13.01667 | 0.31604 | 0.80513 | **0.88828** | **0.00000** |
| HT_ML | HP_IS | 0.529510 | 1.38594 | 13.08673 | 0.29916 | 0.81153 | **0.88423** | **0.00000** |
| HT_ML | HP_IM | 0.566720 | 1.36782 | 13.16883 | 0.26442 | 0.80905 | **0.88162** | **0.00000** |
| HT_ML | HP_CS | 0.598780 | 1.78549 | 13.32313 | 0.24196 | 0.76968 | **0.87787** | **0.00000** |
| HT_ML | HP_AS | 0.648760 | 2.33530 | 12.85238 | 0.20761 | 0.70517 | **0.81846** | **0.00000** |
| HT_ML | HP_AM | 0.633960 | 1.62043 | 13.29853 | 0.22419 | 0.78852 | **0.87368** | **0.00000** |
| HT_MDS | HT_IS | 0.696630 | 1.48332 | 1.57391 | 0.06455 | 0.06029 | 0.06853 | 0.29492 |
| HT_MDS | HT_AS | 0.756590 | 1.52572 | 1.52500 | 0.03588 | 0.03706 | 0.01932 | 0.51465 |
| HT_MDS | HT_AM | 0.784270 | 1.95873 | 1.84800 | 0.02652 | 0.03497 | 0.02718 | 0.40234 |
| HT_MDS | HP_TS | 0.799100 | 2.08582 | 12.29167 | 0.08597 | 0.68869 | **0.84605** | **0.00000** |
| HT_MDS | HP_TO | 0.559730 | 0.91412 | 13.53103 | 0.19061 | 0.84770 | **0.92014** | **0.00000** |
| HT_MDS | HP_TM | 0.702000 | 1.19742 | 13.50000 | 0.13807 | 0.82574 | **0.90676** | **0.00000** |
| HT_MDS | HP_ML | 0.468350 | 0.81497 | 13.58235 | 0.22765 | 0.85243 | **0.92422** | **0.00000** |
| HT_MDS | HP_MDS | 0.565900 | 0.80039 | 13.36000 | 0.21833 | 0.88282 | **0.92845** | **0.00000** |
| HT_MDS | HP_IS | 0.566880 | 0.82456 | 13.42857 | 0.18972 | 0.86271 | **0.92429** | **0.00000** |
| HT_MDS | HP_IM | 0.609800 | 0.86709 | 13.53333 | 0.15573 | 0.84568 | **0.92163** | **0.00000** |
| HT_MDS | HP_CS | 0.700420 | 1.07977 | 13.67143 | 0.16063 | 0.86179 | **0.91744** | **0.00000** |
| HT_MDS | HP_AS | 0.748090 | 1.97232 | 13.13000 | 0.11612 | 0.73292 | **0.86026** | **0.00000** |
| HT_MDS | HP_AM | 0.709570 | 1.09951 | 13.64615 | 0.12639 | 0.82965 | **0.91343** | **0.00000** |
| HT_IS | HT_AS | 0.683460 | 1.53999 | 1.49819 | -0.00495 | 0.00818 | -0.02748 | 0.71387 |
| HT_IS | HT_AM | 0.704620 | 1.85543 | 1.83826 | 0.00059 | 0.02006 | -0.00182 | 0.70117 |
| HT_IS | HP_TS | 0.713120 | 1.94517 | 12.63587 | 0.16559 | 0.74311 | **0.84678** | **0.00000** |
| HT_IS | HP_TO | 0.547530 | 1.07992 | 13.89655 | 0.28473 | 0.85915 | **0.91909** | **0.00000** |
| HT_IS | HP_TM | 0.642610 | 1.30717 | 13.82016 | 0.21747 | 0.83418 | **0.90575** | **0.00000** |
| HT_IS | HP_ML | 0.479720 | 0.98884 | 13.92583 | 0.33024 | 0.86713 | **0.92294** | **0.00000** |
| HT_IS | HP_MDS | 0.548830 | 1.03527 | 13.71087 | 0.29408 | 0.86521 | **0.92709** | **0.00000** |
| HT_IS | HP_IS | 0.552450 | 1.01644 | 13.78106 | 0.28229 | 0.86638 | **0.92305** | **0.00000** |
| HT_IS | HP_IM | 0.586020 | 1.03196 | 13.86561 | 0.25091 | 0.86197 | **0.92035** | **0.00000** |
| HT_IS | HP_CS | 0.627250 | 1.25458 | 14.03106 | 0.22189 | 0.83614 | **0.91643** | **0.00000** |
| HT_IS | HP_AS | 0.670290 | 1.85290 | 13.49130 | 0.19019 | 0.76732 | **0.86075** | **0.00000** |
| HT_IS | HP_AM | 0.653280 | 1.22627 | 13.99498 | 0.20955 | 0.84437 | **0.91246** | **0.00000** |
| HT_AS | HT_AM | 0.744420 | 1.87726 | 1.87667 | 0.01065 | 0.02218 | 0.00297 | 0.50586 |
| HT_AS | HP_TS | 0.753620 | 1.96558 | 12.59549 | 0.13794 | 0.73981 | **0.84395** | **0.00000** |
| HT_AS | HP_TO | 0.587290 | 1.11473 | 13.85632 | 0.25213 | 0.85502 | **0.91673** | **0.00000** |
| HT_AS | HP_TM | 0.686730 | 1.34234 | 13.78030 | 0.18642 | 0.82932 | **0.90334** | **0.00000** |
| HT_AS | HP_ML | 0.517050 | 1.02222 | 13.88603 | 0.29648 | 0.86343 | **0.92060** | **0.00000** |
| HT_AS | HP_MDS | 0.597500 | 1.07822 | 13.67083 | 0.25744 | 0.85914 | **0.92472** | **0.00000** |
| HT_AS | HP_IS | 0.592930 | 1.05314 | 13.74107 | 0.24949 | 0.86187 | **0.92068** | **0.00000** |
| HT_AS | HP_IM | 0.622050 | 1.06517 | 13.82576 | 0.22150 | 0.85826 | **0.91799** | **0.00000** |
| HT_AS | HP_CS | 0.682200 | 1.29854 | 13.99107 | 0.18794 | 0.82882 | **0.91409** | **0.00000** |
| HT_AS | HP_AS | 0.715920 | 1.87726 | 13.43958 | 0.15690 | 0.76293 | **0.85802** | **0.00000** |
| HT_AS | HP_AM | 0.693320 | 1.26024 | 13.95192 | 0.18024 | 0.84013 | **0.91008** | **0.00000** |
| HT_AM | HP_TS | 0.772760 | 2.26590 | 11.99333 | 0.11388 | 0.69305 | **0.81091** | **0.00000** |
| HT_AM | HP_TO | 0.607830 | 1.40300 | 13.15034 | 0.22324 | 0.81242 | **0.88927** | **0.00000** |
| HT_AM | HP_TM | 0.708300 | 1.66869 | 13.09273 | 0.16075 | 0.78210 | **0.87518** | **0.00000** |
| HT_AM | HP_ML | 0.537010 | 1.28762 | 13.18471 | 0.26588 | 0.82333 | **0.89345** | **0.00000** |
| HT_AM | HP_MDS | 0.622310 | 1.42495 | 12.97400 | 0.22633 | 0.80879 | **0.89738** | **0.00000** |
| HT_AM | HP_IS | 0.613780 | 1.34801 | 13.04000 | 0.22068 | 0.81825 | **0.89323** | **0.00000** |
| HT_AM | HP_IM | 0.640430 | 1.33441 | 13.12606 | 0.19532 | 0.81762 | **0.89059** | **0.00000** |
| HT_AM | HP_CS | 0.708840 | 1.69297 | 13.28571 | 0.16069 | 0.77151 | **0.88678** | **0.00000** |
| HT_AM | HP_AS | 0.737840 | 2.20624 | 12.80600 | 0.13851 | 0.71474 | **0.82740** | **0.00000** |
| HT_AM | HP_AM | 0.712920 | 1.56261 | 13.25077 | 0.15564 | 0.79630 | **0.88250** | **0.00000** |
| HP_TS | HP_TO | 0.613310 | 1.47404 | 1.75144 | 0.02856 | 0.08424 | **0.11466** | **0.00000** |
| HP_TS | HP_TM | 0.717090 | 1.75632 | 1.93182 | 0.01062 | 0.07602 | **0.10510** | **0.00000** |
| HP_TS | HP_ML | 0.540670 | 1.35055 | 1.69118 | 0.04738 | 0.08553 | **0.11343** | **0.00000** |
| HP_TS | HP_MDS | 0.629380 | 1.51103 | 1.57917 | 0.02750 | 0.06857 | **0.09706** | **0.00000** |
| HP_TS | HP_IS | 0.619500 | 1.41936 | 1.65179 | 0.02256 | 0.07593 | **0.09993** | **0.00000** |
| HP_TS | HP_IM | 0.646110 | 1.39927 | 1.70581 | 0.01780 | 0.07847 | **0.10272** | **0.00000** |
| HP_TS | HP_CS | 0.719710 | 1.79968 | 1.80060 | 0.00576 | 0.07080 | **0.11210** | **0.00391** |
| HP_TS | HP_AS | 0.747800 | 2.31007 | 2.39583 | 0.00007 | 0.04229 | 0.03798 | 0.40039 |
| HP_TS | HP_AM | 0.721050 | 1.64111 | 1.84295 | 0.00166 | 0.07128 | **0.09628** | **0.00000** |
| HP_TO | HP_TM | 0.547560 | 0.89262 | 0.93574 | 0.00815 | 0.03123 | 0.01994 | 0.26562 |
| HP_TO | HP_ML | 0.420570 | 0.68357 | 0.68661 | -0.00218 | 0.01557 | -0.00150 | 0.51953 |
| HP_TO | HP_MDS | 0.462060 | 0.63710 | 0.60862 | -0.01218 | 0.01509 | -0.00916 | 0.83203 |
| HP_TO | HP_IS | 0.478370 | 0.67613 | 0.67365 | -0.00449 | 0.01679 | -0.00203 | 0.45508 |
| HP_TO | HP_IM | 0.514850 | 0.71760 | 0.72727 | 0.00181 | 0.02223 | 0.01153 | 0.21387 |
| HP_TO | HP_CS | 0.515070 | 0.77025 | 0.77833 | -0.00557 | 0.01986 | -0.01120 | 0.24121 |
| HP_TO | HP_AS | 0.567330 | 1.35461 | 1.48621 | 0.00741 | 0.02263 | -0.00467 | 0.47461 |
| HP_TO | HP_AM | 0.564430 | 0.85070 | 0.85809 | 0.00498 | 0.01935 | 0.00097 | 0.16113 |
| HP_TM | HP_ML | 0.478440 | 0.81664 | 0.87701 | 0.01620 | 0.02686 | 0.01278 | 0.60840 |
| HP_TM | HP_MDS | 0.548900 | 0.80678 | 0.81818 | 0.00383 | 0.03924 | 0.03157 | 0.29102 |
| HP_TM | HP_IS | 0.552590 | 0.82413 | 0.87987 | 0.00609 | 0.03752 | 0.03034 | 0.39648 |
| HP_TM | HP_IM | 0.586800 | 0.85741 | 0.88843 | -0.01005 | 0.01539 | -0.00970 | 0.79102 |
| HP_TM | HP_CS | 0.629790 | 0.99410 | 0.99351 | -0.00202 | 0.04027 | 0.02849 | 0.44824 |
| HP_TM | HP_AS | 0.673560 | 1.64387 | 1.67727 | -0.00005 | 0.02701 | 0.00356 | 0.58789 |
| HP_TM | HP_AM | 0.655710 | 1.03032 | 1.05594 | 0.00205 | 0.03132 | 0.02023 | 0.46289 |
| HP_ML | HP_MDS | 0.398730 | 0.58196 | 0.55588 | -0.00833 | 0.01196 | -0.01266 | 0.58887 |
| HP_ML | HP_IS | 0.422360 | 0.62496 | 0.61765 | -0.00030 | 0.01148 | -0.00986 | 0.96973 |
| HP_ML | HP_IM | 0.460400 | 0.66715 | 0.66221 | 0.00374 | 0.01111 | -0.00816 | 0.72754 |
| HP_ML | HP_CS | 0.437080 | 0.69435 | 0.71849 | -0.00140 | 0.01346 | -0.02405 | 0.90527 |
| HP_ML | HP_AS | 0.493470 | 1.23303 | 1.43235 | 0.02016 | 0.02167 | -0.00664 | 0.40918 |
| HP_ML | HP_AM | 0.498740 | 0.78327 | 0.79638 | 0.01752 | 0.01195 | -0.01207 | 0.86426 |
| HP_MDS | HP_IS | 0.465370 | 0.56043 | 0.53571 | -0.01571 | 0.00769 | -0.02721 | 0.59082 |
| HP_MDS | HP_IM | 0.509890 | 0.61985 | 0.58636 | -0.00745 | 0.01168 | -0.01331 | 0.71875 |
| HP_MDS | HP_CS | 0.507020 | 0.63182 | 0.66429 | -0.01111 | 0.03473 | 0.00296 | 0.11816 |
| HP_MDS | HP_AS | 0.573680 | 1.36842 | 1.36500 | 0.00762 | 0.02439 | -0.00251 | 0.68945 |
| HP_MDS | HP_AM | 0.569250 | 0.76412 | 0.72308 | 0.00246 | 0.01432 | -0.01313 | 0.49707 |
| HP_IS | HP_IM | 0.518330 | 0.65858 | 0.65368 | -0.00531 | 0.01550 | -0.00200 | 0.54492 |
| HP_IS | HP_CS | 0.520300 | 0.68580 | 0.71939 | -0.00757 | 0.02462 | -0.00521 | 0.73535 |
| HP_IS | HP_AS | 0.573030 | 1.29289 | 1.42143 | 0.00467 | 0.02283 | -0.00550 | 0.40918 |
| HP_IS | HP_AM | 0.569390 | 0.78650 | 0.78022 | -0.00140 | 0.01086 | -0.01626 | 0.78906 |
| HP_IM | HP_CS | 0.564630 | 0.73946 | 0.77489 | -0.00304 | 0.02779 | 0.01017 | 0.23047 |
| HP_IM | HP_AS | 0.606560 | 1.28321 | 1.47879 | 0.00355 | 0.02698 | 0.00384 | 0.34668 |
| HP_IM | HP_AM | 0.599590 | 0.82072 | 0.83566 | 0.00144 | 0.01705 | -0.00163 | 0.64551 |
| HP_CS | HP_AS | 0.664920 | 1.66587 | 1.51071 | -0.01586 | 0.01677 | -0.02022 | 0.93457 |
| HP_CS | HP_AM | 0.644540 | 0.92631 | 0.89011 | -0.01117 | 0.01697 | -0.01678 | 0.89160 |
| HP_AS | HP_AM | 0.682040 | 1.52842 | 1.58846 | -0.01248 | 0.01723 | -0.01371 | 0.63281 |
